# Leveraging artificial intelligence chatbots for anemia prevention: A comparative study of ChatGPT-3.5, copilot, and Gemini outputs against Google Search results

**DOI:** 10.1016/j.pecinn.2025.100390

**Published:** 2025-04-01

**Authors:** Shinya Ito, Emi Furukawa, Tsuyoshi Okuhara, Hiroko Okada, Takahiro Kiuchi

**Affiliations:** a School of Nursing, Kitasato University, 1-15-1, Kitasato, Minami-ku, Sagamihara-city, Kanagawa 252-0373, Japan; b University Hospital Medical Information Network (UMIN) Center, The University of Tokyo Hospital, 7-3-1, Hongo, Bunkyo-ku, Tokyo 113-8655, Japan; c Department of Health Communication, School of Public Health, Graduate School of Medicine, The University of Tokyo, 7-3-1, Hongo, Bunkyo-ku, Tokyo 113-8655, Japan

**Keywords:** Anemia, Patient education, ChatGPT, Copilot, Gemini

## Abstract

**Aim:**

This study evaluated the understandability, actionability, and readability of text on anemia generated by artificial intelligence (AI) chatbots.

**Methods:**

This cross-sectional study compared texts generated by ChatGPT-3.5, Microsoft Copilot, and Google Gemini at three levels: “normal,” “6th grade,” and “PEMAT-P version.” Additionally, texts retrieved from the top eight Google Search results for relevant keywords were included for comparison. All texts were written in Japanese. The Japanese version of the PEMAT-P was used to assess understandability and actionability, while jReadability was used for readability. A systematic comparison was conducted to identify the strengths and weaknesses of each source.

**Results:**

Texts generated by Gemini at the 6th-grade level (*n* = 26, 86.7 %) and PEMAT-P version (*n* = 27, 90.0 %), as well as ChatGPT-3.5 at the normal level (*n* = 21, 80.8 %), achieved significantly higher scores (≥70 %) for understandability and actionability compared to Google Search results (*n* = 17, 25.4 %, *p* < 0.001). For readability, Copilot and Gemini texts demonstrated significantly higher percentages of “very readable” to “somewhat difficult” levels than texts retrieved from Google Search (*p* = 0.000–0.007).

**Innovation:**

This study is the first to objectively and quantitatively evaluate the understandability and actionability of educational materials on anemia prevention. By utilizing PEMAT-P and jReadability, the study demonstrated the superiority of Gemini in terms of understandability and readability through measurable data. This innovative approach highlights the potential of AI chatbots as a novel method for providing public health information and addressing health disparities.

**Conclusion:**

AI-generated texts on anemia were found to be more readable and easier to understand than traditional web-based texts, with Gemini demonstrating the highest level of understandability. Moving forward, improvements in prompts will be necessary to enhance the integration of visual elements that encourage actionable responses in AI chatbots.

## Highlights

• AI-generated text about anemia is easier to understand than text on websites.
• Prompts for the 6th grade version and PEMAT-P improve understanding and readability.
• AI chatbots use few visual aids to support understanding and action.
• AI chatbots can promote understanding of anemia in the general adult population.
• Creating prompts for effective diagrams to improve actionability is a challenge.

## Graphical abstract

**Figure:**
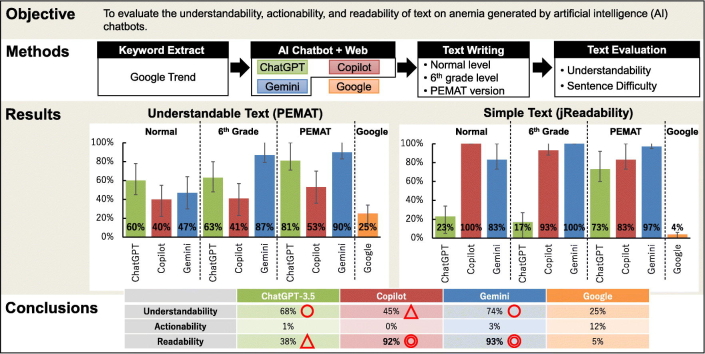

## 1 Introduction

According to the World Health Organization and the Japanese National Health and Nutrition Survey conducted in 2019, 31–32 % of women worldwide and 14.4 % of women in Japan are anemic [1,2]. Iron deficiency anemia (IDA) is the most common cause of anemia and is widely reported to be prevalent in pregnancy, leading to increased maternal and fetal morbidity and mortality [3]. Given these factors, promoting anemia prevention, including improving iron intake through dietary modification, is considered an important issue.

Accessing accurate, understandable, and actionable health information is essential for effective anemia prevention. However, previous studies have highlighted that publicly available health information often exceeds the average literacy level of the general population due to complex terminology and dense content [[4], [5], [6], [7], [8], [9]]. For example, McClure et al. [7] used the Patient Education Materials Assessment Tool for Printable Materials (PEMAT) [10] and the Clear Communication Index [11] to evaluate publicly available educational materials for patients with sickle cell disease. They reported that the literacy level of available patient education materials was above the average literacy level of the adult population in the United States. Studies have also shown that AI-based tools, including large language models (LLMs), can simplify complex health information, making it more understandable and accessible to patients [[12], [13], [14], [15]]. Ito et al. also evaluated the understandability, actionability, and text difficulty of Japanese webpages on anemia, finding that 59 (29.5 %) of materials were easy to understand and 36 (18.0 %) were easy to act on regarding anemia [5].

In addition, the National Workgroup on Cancer and Health [16], the American Medical Association [17], and the Centers for Disease Control and Prevention [18] recommend that patient information materials be readable at a sixth- to eighth-grade level or lower. This readability standard is critical to ensure accessibility for individuals with limited health literacy. However, whether AI chatbots currently represent a useful source of anemia prevention information that aligns with these readability standards remains unclear, as no studies have evaluated the quality of anemia-related texts generated by AI chatbots.

This study focuses on comparing anemia-related educational materials generated by artificial intelligence (AI) chatbots with those retrieved from Google Search. AI chatbots such as ChatGPT, Microsoft Copilot, and Google Gemini have emerged as innovative tools for generating health-related content. These chatbots are capable of producing simplified and structured information, potentially addressing barriers posed by overly complex health communication. In particular, AI chatbots may serve as accessible tools for individuals experiencing mild anemia symptoms, such as dizziness, who might not seek immediate medical attention but still look for initial guidance or general information. While AI chatbots are not yet widely regarded as definitive medical resources, evaluating the understandability of the content they generate is crucial, as they could contribute to early awareness and promote preventive health education. Although prior studies have evaluated the readability, accuracy, and completeness of chatbot outputs [[19], [20], [21]], there is a lack of research specifically comparing AI-generated texts to publicly available information retrieved from Google Search, a widely used resource among the general population.

The purpose of this study was to evaluate the understandability, actionability, and readability of texts about anemia generated by AI chatbots and compare them with information retrieved from Google Search.

## 2 Methods

### 2.1 Information generation and website selection

In this study, we conducted a systematic quantitative content analysis of online materials based on a cross-sectional study. From June 27 to June 28, 2024, webpages were searched using the Google Search engine in Japanese, and text was generated in Japanese by ChatGPT-3.5 (Chat Generative Pretrained Transformer, model 3.5; Open AI, San Francisco, CA, USA), Microsoft Copilot (Microsoft Corporation, Washington, WA, US), and Google Gemini (Alphabet Inc., California, CA, US) AI chatbot. All AI prompts and search keywords were entered in Japanese to ensure consistency with the language of the analyzed webpages. The process for keyword selection was as follows. First, Google Trends was used to search for the top 10 most frequently searched keywords in Japan over the last 5 years, from June 26, 2019, to June 26, 2024.

This section describes the text-generation procedures in Google Search and AI chatbots. First, we describe the procedure for Google. Extracted keywords were entered into Google Search and evaluated in the order of provision by the search engine. Search results were sorted in the order of keyword relevance, so those with lower rankings (i.e., appearing lower on the search results page) were considered to have lower relevance to the keywords. According to a previous study, when the general public uses search engines to search for health-related information, the average number of webpages viewed is approximately eight, and 66 % of users reported viewing fewer than five documents [22]. Based on this, eight webpages were extracted per keyword. Among the search results, videos, advertisements, broken links, sites requiring subscriptions or fees, and professional pages were excluded from evaluation.

Next, we describe the text generation process for the AI chatbot. The following three prompts were used for text generation (Supplement 1). The first was the normal version, “Please tell me about the keyword;” the second was the sixth-grade version, “Please tell me about the keyword at the sixth-grade level;” and the third was the PEMAT-P version, “Please follow the items in the Japanese version of Patient Education Materials Assessment Tool for Printable Materials (PEMAT-P)” [10,23]. The sixth-grade level prompts were chosen because the National Workgroup on Cancer and Health [16], American Medical Association [17], and Centers for Disease Control and Prevention [18] recommend that patient education materials should be written at a sixth- to eighth-grade reading level. This level is considered appropriate for ensuring general adults can understand the content effectively. For the PEMAT-P version, we included prompts designed to meet the criteria of the Patient Education Materials Assessment Tool for Printable Materials (PEMAT-P), which evaluates the understandability and actionability of patient education materials. This condition was added to create materials that are easier to understand and act upon, based on PEMAT-P standards. To ensure that information from the search history did not influence the results, the search history was erased before prompting the chatbots. Because search results can differ even when the same search keyword is used, each AI chatbot was prompted for the same keyword three times. Responses that contained obviously incorrect information regarding search results were excluded from evaluation.

This study was not subjected to ethical review because it did not involve any human subjects or interventions.

### 2.2 Evaluators

A researcher in the field of anemia prevention read each document and independently scored all documents except those for which the content was clearly inappropriate. Next, a physician scored 20 % of randomly selected texts from each condition group using the PEMAT-P [10,23,24]. The PEMAT-P is an index designed to be used by health professionals and medical experts to score information sources. In cases of disagreement, consensus was reached through discussion.

### 2.3 Understandability and actionability

The understandability and actionability of the content provided by each website were evaluated using the Japanese version of PEMAT-P [10,23]. The Japanese version of PEMAT-P consists of 23 items and assesses the following: “content,” “word choice and style,” “use of numbers,” “organization content,” “word choice and style,” “organization,” “layout and design,” and “use of visual aids.” Each item is answered by selecting either 0 (disagree) or 1 (agree). The final PEMAT-P score ranges from 0 % to 100 %, with higher scores indicating greater ease of understanding and actionability. Cutoff values indicating an acceptable result were set at 70 % for both.

### 2.4 Readability

In this study, the jReadability text measurement system was used to quantitatively assess the readability of Japanese text [25,26]. This measurement system calculates the difficulty level of Japanese based on the average length of the text, word difficulty, the percentage of grammatical parts of speech, and the type of characters in each text. Scores range from 0.5 to 6.4, with higher scores indicating that the text is relatively easy to read: 0.5–1.4, “very difficult to read;” 1.5–2.4, “difficult to read;” 2.5–3.4, “somewhat difficult to read;” 3.5–4.4 is “neither;” 4.5–5.4 is “easy to read;” and 5.5–6.4 is “very easy to read.”

### 2.5 Statistical analysis

Three types of statistical analyses were performed in this study. First, within each rating scale, one-factor analysis of variance using Tukey's method was conducted to compare means, the χ^2^ test or Fisher's exact probability test was used to compare proportions, and a Kruskal–Wallis test was used to compare non-normal distributions. Residual analysis, as the posterior analysis of the χ^2^ test, was considered significantly different if the value was greater than 1.96 or less than −1.96. Second, the intraclass correlation coefficient was calculated to compute the level of agreement between evaluators. A significance level of 0.05 was used in this study. All analyses were performed using IBM SPSS for Windows version 25.0 (IBM SPSS Japan, Tokyo, Japan).

## 3 Results

The keywords extracted using Google Trends are shown in Table 1. Ten keywords generated from Google Trends were entered into three AI chatbots (ChatGPT-3.5, Copilot, and Gemini), which generated texts three times at the three reading levels (normal, 6th grade, and PEMAT-P version), for a total of 270 texts. A total of 7 texts (2.6 %) contained obvious errors and were excluded from the analysis: 4 texts (1.5 %) from ChatGPT-3.5 and 3 (1.1 %) from Copilot. Two examples of errors were a lack of explanation of keywords in the generated texts and the fact that criteria values differed significantly. A total of 263 texts (97.4 %) were therefore included in the evaluation. For the web search using Google, the top 8 webpages in search order for 10 keywords were extracted from the webpages that met the relevant criteria. Of the 80 webpages extracted, 67 pages were included in the analysis, excluding duplicate keywords. The intraclass correlation coefficient (ICC) was calculated to quantify the degree of agreement between items, with ICC = 0.57 for PEMAT-P's understandability and ICC = 0.70 for actionability. Regarding AI-generated text, a total of 54 texts (9 conditions × 6 texts) were evaluated by two reviewers. Additionally, 16 texts from Google web pages were assessed, making a total of 70 texts evaluated by the two reviewers.

**Table 1:** Results of keyword searches using Google Trends.

Anemia symptoms
Anemia food
Anemia causes
Anemia menstruation
Anemia tests
Iron deficiency anemia
Aplastic anemia
Anemia values
Anemia treatment
Pregnancy anemia

### 3.1 Understandability and actionability

Table 2, Table 3 show the results of comparisons of scale scores for the AI chatbots and Google Search. Table 4 shows the comparison of PEMAT-P item scores. First, we discuss the percentage of materials that met the cutoff score of ≥70 % in PEMAT-P (Table 2). With regard to understandability, Gemini (*n* = 26, 86.7 %) 6th-grade version, ChatGPT-3.5 (*n* = 21, 80.8 %) and Gemini (*n* = 27, 90.0 %) PEMAT-P-version texts showed significantly higher percentages of scores ≥70 %, while Google Search had a significantly lower percentage of scores ≥70 % (*n* = 17, 25.4 %). With regard to actionability, Google Search had a significantly higher percentage of scores ≥70 % (*n* = 8, 11.9 %).

**Table 2:** Comparison of item scores among artificial intelligence chatbots and web search (categorical variables).

		Artificial Intelligence Chatbot																											Web Search		
		Normal Level									6th Grade Level									PEMAT											
		ChatGPT-3.5			Copilot			Gemini			ChatGPT-3.5			Copilot			Gemini			ChatGPT-3.5			Copilot			Gemini			Google		
		n	%	AR	n	%	AR	n	%	AR	n	%	AR	n	%	AR	n	%	AR	n	%	AR	n	%	AR	n	%	AR	n	%	AR
PEMAT (> 70)																															
	Understandability	18	60.0	0.6	12	40.0	−1.7	14	46.7	−0.9	19	63.3	1.0	11	40.7	−1.5	26	86.7	3.7	21	80.8	2.8	16	53.3	−0.2	27	90.0	4.1	17	25.4	−5.4
	Actionability	0	0.0	−1.1	0	0.0	−1.1	2	6.7	0.9	0	0.0	−1.1	0	0.0	−1.1	0	0.0	−1.1	1	3.8	0.1	0	0.0	−1.1	1	3.3	−0.1	8	11.9	4.1
jReadability difficulty level⁎		7	23.3	−4.3	30	100.0	4.7	25	83.3	2.7	5	16.7	−5.1	25	92.6	3.6	30	100.0	4.7	19	73	1.4	25	83.3	2.7	29	96.6	4.3	3	4.5	−10.4
	Very readable	0	0.0	−1.3	3	10.0	1.4	6	20.0	4.1	0	0.0	−1.3	1	3.7	−0.3	5	16.7	3.2	0	0.0	−1.2	0	0.0	−1.3	1	3.3	−0.4	0	0.0	−2.1
	Readable	0	0.0	−3.2	11	36.7	1.7	12	40.0	2.2	0	0.0	−3.2	8	29.6	0.7	17	56.7	4.4	3	11.5	−1.5	10	33.3	1.3	18	60.0	4.9	0	0.0	−5.1
	Neutral	7	23.3	−1.0	16	53.3	2.7	7	23.3	−1.0	5	16.7	−1.8	16	59.3	3.3	8	26.7	−0.6	16	61.5	3.5	15	50.0	2.3	10	33.3	0.3	3	4.5	−5.3
	Somewhat difficult	18	60.0	3.8	0	0.0	−3.7	5	16.7	−1.6	18	60.0	3.8	2	7.4	−2.6	0	0.0	−3.7	7	26.9	−0.3	5	16.7	−1.6	1	3.3	−3.3	42	62.7	6.6
	Difficult	5	16.7	1.2	0	0.0	−1.9	0	0.0	−1.9	7	23.3	2.5	0	0.0	−1.8	0	0.0	−1.9	0	0.0	−1.8	0	0.0	−1.9	0	0.0	−1.9	22	32.8	6.8

The χ^2^ test was performed to calculate adjusted residuals (ARs); a significant difference was defined as an AR greater than 1.96 or less than −1.96 (*p* < 0.05).

⁎ “Very readable” to “Somewhat difficult” total is listed.

**Table 3:**
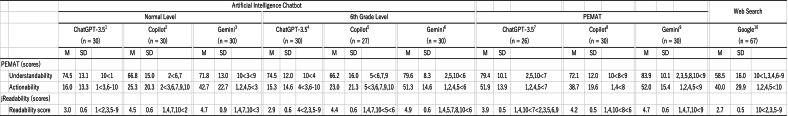
Comparison of scale scores among artificial intelligence chatbots and web search (continuous variables).

ANOVA with a significance level of 5 % was performed, and groups with significant differences are indicated by inequalities.

**Table 4:** PEMAT-P item scores among artificial intelligence chatbots and web search.

		Artificial Intelligence Chatbot																											Web Search		
		Normal Level									6th Grade Level									PEMAT											
		ChatGPT-3.5			Copilot			Gemini			ChatGPT-3.5			Copilot			Gemini			ChatGPT-3.5			Copilot			Gemini			Google		
		n	%	AR	n	%	AR	n	%	AR	n	%	AR	n	%	AR	n	%	AR	n	%	AR	n	%	AR	n	%	AR	n	%	AR
**Understandability**																															
Content																															
1	The material makes its purpose completely evident	29	96.7	1.4	28	93.3	0.7	29	96.7	1.4	28	93.3	0.7	24	88.9	−0.1	28	93.3	0.7	23	88.5	−0.2	28	93.3	0.7	29	96.7	1.4	49	73.1	−4.8
2	The material does not include information or content that distracts from its purpose	30	100.0	1.1	30	100.0	1.1	28	93.3	−0.9	30	100.0	1.1	25	92.6	−1.1	28	93.3	−0.9	26	100.0	1.0	30	100.0	1.1	27	90.0	−2.0	64	95.5	−0.4
Word Choice & Style																															
3	The material uses common, everyday language	16	53.3	−0.8	15	50.0	−1.2	15	50.0	−1.2	20	66.7	0.8	15	55.6	−0.5	24	80.0	2.3	20	76.9	1.8	15	50.0	−1.2	24	80.0	2.3	34	50.7	−1.7
4	Medical terms are defined when they are used	17	56.7	0.1	16	53.3	−0.3	13	43.3	−1.5	21	70.0	1.6	15	55.6	−0.1	27	90.0	3.9	18	69.2	1.4	15	50.0	−0.7	25	83.3	3.2	18	26.9	−5.4
Use of Numbers																															
5	Numbers appearing in the material are clear and easy to understand^1^	1	33.3	0.5	2	22.2	0.1	2	28.6	0.5	1	33.3	0.5	1	16.7	−0.3	2	50.0	1.4	0	0.0	−0.7	2	22.2	0.1	3	50.0	1.8	7	14.0	−1.8
6	The material does not expect the user to perform calculations^1^	30	100.0	1.0	29	96.7	−0.2	30	100.0	1.0	30	100.0	1.0	26	96.3	−0.3	30	100.0	1.0	26	100.0	0.9	30	100.0	1.0	30	100.0	1.0	60	89.6	−4.3
Organization																															
7	The material breaks or “chunks” information into short section		–			–		15	100.0	1.9		–			–		25	100.0	2.6	25	100.0	2.6	14	100.0	1.8	30	100.0	2.9	26	46.4	−8.4
8	The material's sections have informative headers		–			–		15	100.0	1.0		–			–		24	96.0	0.3	25	100.0	1.3	14	100.0	0.9	29	96.7	0.6	49	87.5	−2.9
9	The material presents information in a logical sequence	30	100.0	0.9	30	100.0	0.9	30	100.0	0.9	29	96.7	−0.3	26	96.3	−0.5	29	96.7	−0.3	26	100.0	0.8	29	96.7	−0.3	29	96.7	−0.3	64	95.5	−1.2
10	The material provides a summary		–			–		1	6.7	−2.5		–		0	0.0	−0.8	8	30.8	−0.6	11	44.0	0.9	2	14.3	−1.8	25	86.2	6.2	13	23.2	−2.5
Layout & Design																															
11	The material uses visual cues to draw attention to key points	28	93.3	1.3	16	53.3	−5.1	29	96.7	1.9	22	73.3	−1.9	15	55.6	−4.5	29	96.7	1.9	26	100.0	2.2	30	100.0	2.4	30	100.0	2.4	56	83.6	−0.4
Use of Visual Aids																															
12	The material uses visual aids whenever they could make content more easily understood	0	0.0	−1.6	0	0.0	−1.6	2	6.7	−0.2	0	0.0	−1.6	3	11.1	0.7	0	0.0	−1.6	0	0.0	−1.5	0	0.0	−1.6	0	0.0	−1.6	20	30.3	7.8
13	The material's visual aids reinforce rather than distract from the content		–			–		2	100.0	1.8		–		1	33.3	−0.2		–			–			–		0	0.0	−0.8	18	38.3	−0.6
14	The material's visual aids have clear titles or captions		–			–		2	100.0	1.9		–		1	33.3	−0.2		–			–			–		1	100.0	1.3	16	34.0	−1.6
15	The material uses illustrations and photographs that are clear and uncluttered		–			–		1	50.0	−0.5		–		2	66.7	0.0		–			–			–		0	0.0	−1.4	32	68.1	0.9
16	The material uses simple tables with short and clear row and column headings^1^		–			–			–			–			–			–		3	100.0	0.9		–		3	100.0	0.9	10	71.4	−1.5
**Actionability**																															
17	The material clearly identifies at least one action the user can take	20	66.7	−1.8	20	66.7	−1.8	27	90.0	1.5	19	63.3	−2.3	17	63.0	−2.2	29	96.7	2.5	26	100.0	2.7	25	83.3	0.6	29	96.7	2.5	50	74.6	−1.1
18	The material addresses the user directly when describing actions	3	10.0	−5.8	16	53.3	−0.7	21	70.0	1.2	3	10.0	−5.8	10	37.0	−2.5	27	90.0	3.6	21	80.8	2.3	24	80.0	2.4	28	93.3	4.0	43	64.2	0.9
19	The material breaks down any action into manageable, explicit steps	1	3.3	−3.8	2	6.7	−3.4	13	43.3	1.0	1	3.3	−3.8	4	14.8	−2.3	20	66.7	3.8	20	76.9	4.7	9	30.0	−0.6	20	66.7	3.8	25	37.3	0.5
20	The material provides a tangible tool whenever it could help the user take action	0	0.0	−1.3	0	0.0	−1.3	2	6.7	0.5	0	0.0	−1.3	0	0.0	−1.2	1	3.3	−0.4	1	3.8	−0.2	0	0.0	−1.3	1	3.3	−0.4	11	16.4	4.9
21	The material provides simple instructions or examples of how to perform calculations^1^		–			–			–			–			–			–			–			–			–			–	
22	The material explains how to use the charts, graphs, tables, or diagrams to take actions^1^	0	0.0	−0.6	0	0.0	−0.6	1	3.3	1.1	0	0.0	−0.6	0	0.0	−0.6	0	0.0	−0.6	0	0.0	−0.6	0	0.0	−0.6	0	0.0	−0.6	3	4.5	2.7
23	The material uses visual aids whenever they could make it easier to act on the instructions	0	0.0	−0.8	0	0.0	−0.8	1	3.3	0.5	0	0.0	−0.8	0	0.0	−0.8	0	0.0	−0.8	0	0.0	−0.8	0	0.0	−0.8	0	0.0	−0.8	6	9.0	4.3

1 Including not applicable to the response method. AR = adjusted residual; Significant difference if AR is greater than 1.96 or less than −1.96 (p < 0.05).

Next, we discuss the results of comparisons among PEMAT-P scale scores (Table 3). For understandability, PEMAT-P-version texts from Gemini showed the highest scale scores (M = 83.9, SD = 10.1), significantly higher than those of the normal-level Copilot (M = 66.8, SD = 15.0) and Gemini (M = 71.8, SD = 13.0), 6th-grade Copilot (M = 66.2, SD = 16.0), PEMAT-P-version Copilot (M = 72.1, SD = 12.0), and Google Search (M = 58.5, SD = 16.0). For actionability, PEMAT-P-version texts from ChatGPT-3.5 (M = 51.9, SD = 13.9) and Gemini (M = 52.0, SD = 15.4) had the highest scale scores. However, no group had an average score above the cutoff of 70 %.

Table 4 shows the results of the PEMAT-P item score comparisons among AI chatbots and Google Search. The website text by Google had a significantly lower percentage of scores ≥70 % on six of the items related to understandability than the AI-generated texts.

### 3.2 Readability

For the jReadability score, normal-grade Copilot (*n* = 30, 100.0 %) and Gemini (*n* = 25, 83.3 %), 6th-grade Copilot (n = 25, 92.6 %) and Gemini (n = 30, 100.0 %), and PEMAT-P-version Copilot (n = 25, 83.3 %) and Gemini (*n* = 29, 96.6 %) had significantly higher percentages of “very readable” to “somewhat difficult” responses. Conversely, ChatGPT-3.5 at the normal level (*n* = 7, 23.3 %) and 6th-grade level (*n* = 5, 16.7 %) and Web Search (n = 3, 4.5 %) had significantly lower percentages of “very readable” to “somewhat difficult” responses. Comparisons of mean scores for jReadability difficulty (Table 3) showed that scores were highest for Normal (M = 4.7, SD = 0.9), 6th-grade (M = 4.9, SD = 0.6), and PEMAT-P (M = 4.7, SD = 0.6) versions of Gemini.

## 4 Discussion

The purpose of this study was to investigate whether AI-generated texts could provide a useful resource on anemia prevention for the general public. The results suggest that AI-generated text about anemia is easier to understand and less difficult to comprehend than text on websites. Specifically, AI-generated texts were superior, in that they had a clear purpose for the text, defined medical terms, broke the information into shorter sections, and included a summary. On the other hand, AI chatbots rarely used visual aids effectively to promote understanding or action, even when prompts instructed them to generate visual aids. In terms of actionability, webpage text was superior to AI-generated text, but only 8 webpage texts (11.9 %) were easy to act on, suggesting that both AI text and web materials have issues with ease of actionability. These results suggest that AI-generated texts may be useful materials for preventing anemia among the general public because they are in easily understandable, less difficult Japanese. On the other hand, effective methods of generating prompts must be considered in order to present figures and tables that are easy to understand and act upon. Although this study focused on anemia, the methodologies employed, including the systematic evaluation of understandability, actionability, and readability, are broadly applicable to other health conditions. For example, diseases such as diabetes or hypertension often face similar challenges in patient education. Applying these methods to other conditions could reveal new insights into improving the accessibility and usability of health information.

A comparison of anemia-related texts generated by the three AI chatbots showed that Gemini was the easiest to understand, and provided Japanese text at the least difficult reading level. One possible explanation for the superior readability and understandability of AI-generated texts is that they are generated following a structured pattern, which ensures clarity through defined medical terms, segmented sections, and summaries. In particular, Gemini appears to have incorporated action-oriented instructions as part of its generation pattern, which may have contributed to its higher actionability. In addition, few texts showed high actionability. Specifically, texts generated by Gemini were superior in using everyday terms, defining medical terms, breaking texts into short sections, providing summaries, showing what the user could do, and providing direct recommendations regarding actions to be taken by the reader. The results of these comparisons among AI chatbots were partially consistent with the results of previous studies [[27], [28], [29], [30]]. A study by Tepe and Emekli used ChatGPT-4.0, Copilot, and Google BARD to simplify radiology reports and reported that BARD was the easiest to understand and the least difficult reading level [27]. A study by Bellinger et al. compared texts generated by ChatGPT, ChatGPT Plus, BARD, and Copilot for the management of common rhinology conditions [28]. They reported ChatGPT Plus and BARD as the easiest to understand, while BARD and Copilot used the lowest text difficulty. Given the differences in comprehensibility and text difficulty among AI chatbots, use of multiple AI chatbots depending on the topic may be important.

The results of the comparison of the three types of prompts show that for Gemini, the PEMAT-P version was easier to understand than the normal version. For ChatGPT-3.5, the PEMAT-P version was also easier to understand than the normal and 6th-grade versions, and the texts were less difficult. The finding that the AI chatbot in this study improved comprehensibility and reduced reading difficulty is partially consistent with the findings of previous studies [29,30]. For example, Dihan et al. used ChatGPT-3.5, ChatGPT-4.0, and Google Bard to assess comprehensibility and text difficulty for patient education materials on pediatric glaucoma, revealing that only ChatGPT-4.0 improved comprehensibility when prompted to provide information at a 6th-grade reading level, but text difficulty was decreased for all AI chatbots [29]. In a study by Shen et al., ChatGPT-3.5 and Google Search were used to generate responses to patient questions about practice guidelines [30]. The resulting text difficulty was lower when responses were prompted to be given at the 6th-grade level than when they were not.

These results suggest that AI chatbots are able to generate texts that are easier to understand and less challenging with the addition of detailed prompts such as those in the PEMAT-P version. On the other hand, the PEMAT-P versions of prompts did not effectively generate illustrations, irrespective of the presence of instructions regarding the generation of illustrations. Specifically, the words “insert illustration here” were seen in the text, but no specific illustration was attached. With regard to the generation of figures and tables, we believe that prompts for generating figures and tables need to be separated from prompts for generating text, and prompts for generating figures and tables need to be instructed independently.

Various limitations to this study were also apparent. First, the prompts presented to the AI chatbots were unable to produce figures or tables that prompted understanding or actionability. Prompts in the PEMAT-P version of this study included instructions such as “Always use visual material when it makes the content easier to understand” and “Always use visual material when it makes it easier to follow the instructions.” However, regarding the insertion of graphs, although text was provided that indicated an insertion point, graphs were seldom actually inserted. Similarly, tables were generated in a few cases, but no row headings were given and the tables were not designed to encourage actionability. Conceivably, to present effective charts that promote understanding and actionability, specific prompts may need to be created for each topic, rather than general instructions.

Additionally, this study focused on two specific types of prompts: a sixth-grade level and a PEMAT-P-based template. While these prompts were chosen to enhance readability and actionability, the study did not explore the effects of other types of prompts, such as specific patient questions or related keywords. Moreover, it remains unclear how the use of different prompts might affect the quality and usability of AI-generated health information when applied to other health conditions or broader contexts. Future studies should investigate the impact of prompt variations to better understand their influence on the generation of effective health information.

Second, because we did not evaluate paid AI chatbots, the results of this study are not necessarily generalizable to paid AI chatbots, which may have superior AI capabilities. However, someone researching anemia information for the first time would be unlikely to register with a paid AI chatbot or read a paid website. Finally, AI chatbots present challenges in terms of information reliability, as incorrect information is occasionally produced.

### 4.1 Innovation

The innovation of this study lies in its pioneering approach as the first to objectively and quantitatively evaluate the understandability, actionability, and readability of anemia-related educational materials generated by AI chatbots. By employing PEMAT-P and jReadability, this study provided numerical evidence of the differences in quality across AI-generated texts and traditional web-based materials. Furthermore, the study demonstrated that AI chatbots like Gemini can generate highly readable and understandable content tailored to the general public. This methodology introduces a new standard for evaluating the quality of health education materials.

In addition, this study highlights the potential of AI chatbots in addressing health disparities. Due to their high accessibility and ability to produce simplified and structured content, AI chatbots represent a promising tool for disseminating health information to underserved populations, including those with limited healthcare access or lower literacy levels. By establishing a foundation for systematic evaluation of AI-generated health information, this study opens new possibilities for improving health communication and addressing inequities in information dissemination.

### 4.2 Conclusion

The results of this study revealed that AI-generated texts on anemia are easier to understand and more readable compared to traditional web-based texts, with Gemini demonstrating the highest levels of understandability and readability. However, challenges such as the lack of actionable visual aids and diagrams were also identified. These findings suggest that AI chatbots hold significant potential as tools for health education on anemia prevention, while emphasizing the need for future optimization of prompts to effectively integrate visual aids that enhance actionability.

## CRediT authorship contribution statement

**Shinya Ito:** Writing – review & editing, Writing – original draft, Visualization, Validation, Software, Resources, Project administration, Methodology, Investigation, Funding acquisition, Formal analysis, Data curation, Conceptualization. **Emi Furukawa:** Writing – review & editing, Validation, Supervision, Methodology, Data curation, Conceptualization. **Tsuyoshi Okuhara:** Writing – review & editing, Validation, Supervision, Conceptualization. **Hiroko Okada:** Writing – review & editing, Validation, Supervision, Methodology, Conceptualization. **Takahiro Kiuchi:** Writing – review & editing, Validation, Supervision, Project administration, Methodology, Conceptualization.

## Informed consent

Not applicable because the study did not involve human subjects.

## Ethical approval

Not applicable because the study did not involve human subjects.

## Funding

This work was supported by the 10.13039/501100001691Japan Society for the Promotion of Science under KAKENHI grant no. JP 24K06389.

## Declaration of competing interest

The authors declare the following financial interests/personal relationships which may be considered as potential competing interests:

Shinya ITO reports financial support was provided by Japan Society for the Promotion of Science. If there are other authors, they declare that they have no known competing financial interests or personal relationships that could have appeared to influence the work reported in this paper.

## Data Availability

The datasets generated and/or analyzed during the current study are available from the corresponding author on reasonable request.

## Appendix A Supplementary data

Supplementary material Image 1
