# Supplementary material for: Leveraging artificial intelligence chatbots for anemia prevention: A comparative study of ChatGPT-3.5, copilot, and Gemini outputs against Google Search results
Source: PEC Innov. 2025 Apr 1;6:100390. doi: 10.1016/j.pecinn.2025.100390 (PMC12020902; doi:10.1016/j.pecinn.2025.100390)
Supplement: Supplementary file 1 — Supplementary material [file mmc1.docx]

**Supplement**

**Prompt for the PEMAT-P version:**

Please tell me about " **KEY WORD** " in 6th grade level Japanese.

Please completely clarify the purpose of the material.

Please do not include information or content that distracts from the purpose of the material.

Please use the common, everyday language of the material.

Please define medical terms when using them.

Please be clear and easy to understand numbers appearing in the material.

Please do not expect the user to perform calculations on the material.

Please break down or "chunk" the information into short sections.

Please have informative headers in the sections of the material.

Please present information in the material in a logical order.

Please provide a summary of the material.

Please use visual cues to draw attention to key points in the material.

Please use visual aids whenever they could make the content of the material easier to understand.

Please have clear titles or captions for visual aids in the material.

Please use illustrations and photographs of the material that are clear and uncluttered.

Please use simple tables with short and clear headings for the rows and columns of the material.

Please clearly identify at least one action the user can take in the material.

Please address the user directly when describing actions in the material.

Please break each action into manageable, explicit steps in the material.

Please provide a tangible tool whenever it could help the user take action in the material.

Please provide simple instructions or examples of how to perform calculations in the material.

Please explain in the material how to use the charts, graphs, tables, or diagrams to take action.

Please use visual aids in the material whenever they could make it easier to act on the instructions.
